# Wilms tumor with Mulibrey Nanism: A case report and review of literature

**DOI:** 10.1002/cnr2.1512

**Published:** 2021-07-26

**Authors:** Karthik Upasana, Dhwanee Thakkar, Dheeraj Gautam, Manvinder Singh Sachdev, Anjali Yadav, Rohit Kapoor, Veena Raghunathan, Maninder Singh Dhaliwal, Kartikeya Bhargava, Sandhya Nair, Jaiprakash Sharma, Neha Rastogi, Satya Prakash Yadav

**Affiliations:** ^1^ Pediatric Hematology Oncology and Bone Marrow Transplant Unit, Cancer Institute Medanta The Medicity Hospital Gurgaon Haryana India; ^2^ Department of Pathology Medanta The Medicity Gurgaon Haryana India; ^3^ Department of Pediatric Cardiology Medanta The Medicity Gurgaon Haryana India; ^4^ Pediatric Intensive Care Unit, Department of Pediatrics Medanta The Medicity Gurgaon Haryana India; ^5^ Department of Electrophysiology and Pacing, Heart Institute Medanta The Medicity Gurgaon Haryana India; ^6^ MedGenome Labs Ltd. Bangalore Karnataka India; ^7^ Division of Radiology Medanta The Medicity Gurgaon Haryana India

**Keywords:** atrial flutter, atrial septal defect, Mulibrey Nanism, Wilms tumor

## Abstract

**Background:**

Mulibrey‐Nanism (*Mu*scle‐*li*ver‐*br*ain‐*e*ye Nanism = dwarfism; MUL) is a rare genetic syndrome. The underlying TRIM37 mutation predisposes these children to develop tumors frequently. In the largest published series of MUL, 8% patients were reported to develop Wilms tumor (WT). The published literature lacks data regarding the best treatment protocol and outcome of this cohort of children with WT and MUL. We report here a 2‐year‐old boy with WT and MUL and present a review of literature on WT in MUL.

**Case:**

Our patient had associated cardiac problems of atrial septal defect, atrial flutter and an episode of sudden cardiac arrest. We managed him successfully with chemotherapy, surgery and multi‐speciality care. He is alive and in remission at follow‐up of 6 months.

**Conclusion:**

A total of 14 cases (including present case) of WT have been reported in MUL and treatment details were available for six cases. They were managed primarily with surgery, chemotherapy with/without radiotherapy, and all achieved remission. The outcome data is available only for two cases, one has been followed up till 15 years post treatment for WT and other is our patient.

**Abbreviations**
AF: atrial flutter
ASD: atrial septal defect
MUL: Mulibrey Nanism
WT: Wilms tumor

## 1 INTRODUCTION

Mulibrey‐Nanism (MUL) (*Mu*scle‐*li*ver‐*br*ain‐*e*ye Nanism = dwarfism), is an autosomal recessive disorder caused by mutations in the TRIM37 gene.^1^ TRIM37 is a 130 kDa protein located in the peroxisomes and expressed in several tissues of the body.^2^ It is implicated in various biological processes, such as post‐translational modifications, signal transduction, DNA repair, immunological signaling, autophagy and oncogenesis.^3^ The most frequently encountered “Fin‐major mutation” *c*.*493‐2A>G* has been reported in Finnish patients probably due to a founder effect.^3^ Non‐Finnish MUL patients have been reported with other mutations.^4^ The syndrome is characterized by growth failure of perinatal onset, dysmorphic facies, muscular dystonia, venous congestion secondary to constrictive pericarditis and yellowish dots in fundi. They have an increased tendency to develop cystic and benign adenomatous lesions in various parts of the body, cutaneous nevi flammi, gonadal dysfunction especially in females, type II diabetes mellitus, fibrous dysplasia and malignancy, mainly Wilms tumor (WT).^5^, ^6^ There are very few reports of WT in MUL and the published literature lacks data regarding the best treatment protocol and outcome of this cohort. Here we report a child of WT with MUL and provide a review of literature.

## 2 METHOD

We reviewed medical records of our case of a child with MUL and WT and looked at treatment details, complications and outcome. We performed a review of the literature for the similar cases. MED‐LINE/PubMed/Google/Google Scholar/EMBASE/Cochrane were searched from inception to March 2021, and the following terms were used for data searching: “Mulibrey Nanism” OR “TRIM37” AND “Wilms tumor”. Full‐text manuscripts or abstracts, published in English language peer‐reviewed journal were included. Articles published in languages other than English for which formal translation was not available were excluded. Free full‐text articles available at https://pubmed.ncbi.nlm.nih and selected full‐text articles indexed in Web of science or Scopus are downloaded using the EBSCO discovery services.

## 3 RESULTS

A 2‐year‐old boy presented with incidentally detected mass in left side of his abdomen. Computerized tomography (CT) scan of abdomen showed a heterogeneously enhancing, solid cystic lesion measuring 59 × 56 × 82 mm in the upper pole of the left kidney. Liver was also enlarged with hypodense lesions in segments IVB/V and VI. CT chest showed no evidence of metastasis. Renal and liver biopsies were done at another center. Review of renal biopsy confirmed the diagnosis of WT. Liver biopsy showed no tumor. He was born of non‐consanguineous parents with birth weight of 2800 g (between −2 and 0, *Z* score as per WHO growth chart) and length of 49 cm (between −2 and 0, *Z* score as per WHO growth chart). On examination, child had a triangular face, low nasal bridge, high broad forehead, low set ears, a high‐pitched voice, distended abdomen, left sided ballotable renal mass and hepatomegaly. Neurological examination revealed mild hypotonia and normal higher mental functions. His present height was 87 cm (15th–50th centile as per WHO growth chart) and weight was 10 kg (third centile as per WHO growth chart). Echocardiography showed moderate size secundum atrial septal defect (ASD) with bidirectional shunt predominantly left to right and restrictive filling of right ventricle as evidenced by tissue doppler imaging. Final diagnosis was considered WT stage 4 due to multiple nodules seen in liver. He was started on neo‐adjuvant chemotherapy as per SIOP2001 protocol.

He received five doses of weekly injection vincristine and one dose of injection actinomycin‐D. Doxorubicin was not given in view right ventricular dysfunction and was replaced with cyclophosphamide. After week 5 of chemotherapy child was admitted with febrile neutropenia and started on intravenous antibiotics. On the second day of this admission he complained of restlessness, vomiting and hypotension, closely followed by generalized tonic–clonic seizures and went into sudden cardiac arrest. Child was revived after cardiopulmonary resuscitation. As he was still in coma and had jerky respiration so was intubated and ventilated. His condition improved over next 48 h and he was extubated. Electroencephalogram was normal. Magnetic resonance imaging of brain was normal except for presence of a choroid plexus/xanthogranulomatous cyst in left lateral ventricle and a shallow and J shaped Sella turcica. He developed hoarseness of voice which was possibly due to vincristine and intubation. Subsequently, he developed an episode of paroxysmal tachycardia with heart rate >220/min, diagnosed as atrial flutter (AF) on electrocardiogram. It was aborted with injection amiodarone. Gradually his voice returned and he could swallow food. In view of cough, he was tested for SARS‐COV‐2 by PCR and it was detected to be positive. He was shifted to COVID‐19 care ward where he had a second episode of atrial flutter and required to be shifted to intensive care unit again. He was managed successfully for AF and his cardiac rhythm reverted to normal. Once he was negative for COVID‐19 he was discharged.

In view of child not tolerating chemotherapy well, having abnormal facies, cardiac anomaly, hepatomegaly and abnormal findings in MRI brain; it was suspected that child has some underlying syndrome. So, a karyotyping with standard 400–450 G banding technique was performed and it was reported as normal. Mitomycin C sensitivity testing was done on 72 h PHA stimulated unsynchronized cultures to rule of Fanconi Anemia and was reported as normal. Finally a clinical exome sequencing using selective capture and sequencing of the protein coding regions of the genome/genes was performed. The library was sequenced to mean >80–100× coverage on Illumina sequencing platform. The sequences obtained was aligned to human reference genome (GRCh38.p13) using Sentieon aligner and analyzed using Sentieon for removing duplicates, recalibration and re‐alignment of indels.^7^ Sentieon haplotype caller was used to identify variants which are relevant to the clinical indication. Gene annotation of the variants was performed using VEP program^8^ against the Ensembl release 99 human gene model.^9^ In addition to SNVs and small indels, copy number variants (CNVs) were detected from targeted sequence data using the Exome Depth (v1.1.10) method.^10^ Clinically relevant mutations were annotated using published variants in literature and a set of diseases databases—ClinVar, OMIM (updated on February 20, 2020), GWAS, HGMD (v2019.4) and SwissVar.^11^, ^12^, ^13^, ^14^ Common variants were filtered based on allele frequency in 1000 Genome Phase 3, gnomAD (v2.1), EVS, dbSNP (v151), 1000 Japanese Genome and our internal Indian population database. Non‐synonymous variants effect was calculated using multiple algorithms such as PolyPhen‐2, SIFT, MutationTaster2 and LRT. Only non‐synonymous and splice site variants found in the clinical exome panel consisting of 6670 genes were used for clinical interpretation. Sequencing was reported to show the presence of heterozygous nonsense variation in exon 15 of the TRIM37 gene variant *c*.*1336C>T* (chr17: g. 59049372 G>A; Depth: 130×) resulting in a stop codon and premature truncation of the protein at codon 446 (p. Arg446Ter). This mutation was reported as a pathogenic variant for Mulibrey‐Nanism. Another heterozygous missense variation in exon 2 of the TRIM37 gene variant *c*.*80T>A* (chr17: g.59104336A>T; Depth: 94×) that results in the amino acid substitution of Glutamine for Leucine at codon 27 (p. Leu27Gln; ENST00000262294.12) was detected but was analyzed to be of unknown significance.

The genomic DNA samples of the parents were analyzed for the reported variants in TRIM37 gene using PCR followed by DNA sequencing. Mother was negative for both c.80T>A and c.1336C>T mutations and father was heterozygous for c.80T>A and negative c.1336C>T. Although the c.80T>A has been reported to be of unknown significance, parental testing has proven that it is inherited from father and the other known mutation is de novo. The child satisfied the clinical criteria for diagnosis of MUL due to presence three major criteria (small for gestational age at birth, J‐shaped Sella turcica on MRI, typical facies) and two minor criteria (high pitched voice and hepatomegaly). Along with heterozygous pathogentic mutation, the second heterozygous novel mutation could possibly be considered deleterious causing MUL in him.

**FIGURE 1:**
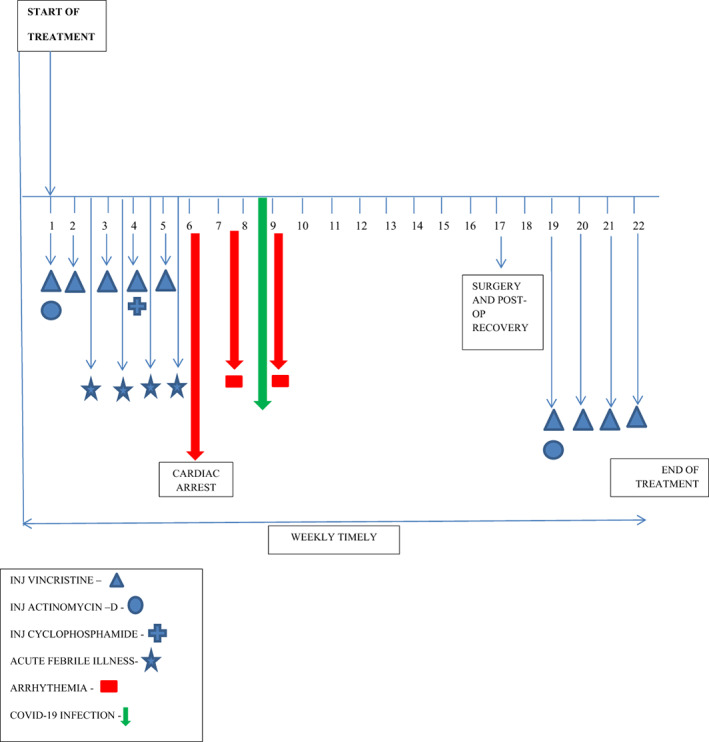
Treatment details and complications

Positron emission tomography‐CT done after completion of 5‐weeks of chemotherapy showed reduction in size of renal tumor with multiple non‐FDG avid liver lesions. CT chest was suggestive of minimal cardiomegaly with basal areas of parietal pericardium showing evidence of early calcification. He underwent nephrectomy and histopathology was suggestive of favorable histology WT (blastemal 40%, epithelial 30% and stromal 30%). Review of tissue block of liver lesions was not suggestive of WT metastasis and a possibility of benign hamartomas was kept. Post operatively tumor was classified as stage‐I. Figure 1 illustrates his treatment course. He was given further four weekly doses of vincristine at 50% dose in view of previous toxicity and a single dose of actinomycin‐D. At present, he is well 6‐months after finishing his planned chemotherapy. He underwent fundus examination which was within normal limits. His endocrinology work‐up showed presence of hypothyroidism which was treated with thyroxine. The patient is currently on oral antiarrhythmic (Metoprolol and Amiodarone) and has not experienced further episodes of atrial flutter.

## 4 REVIEW OF LITERATURE

**FIGURE 2:**
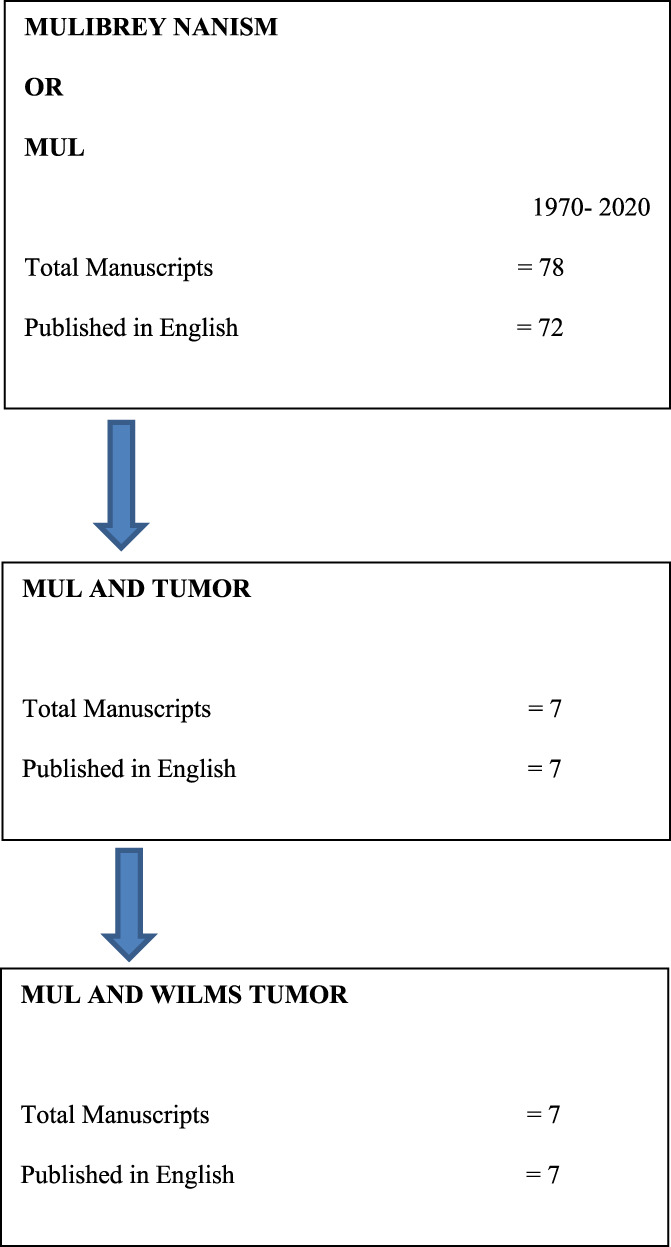
Algorithm for review of literature

On review of literature, we found a total of 14 cases including one case of ours.^6^, ^15^, ^16^, ^17^, ^18^, ^19^ The algorithm used for review of literature is shown in Figure 2. Table 1 show details of all the cases. The mean age of the subjects was 19 months (Male: Female ratio 1:1). Other than our case, histopathology detail was available for only one other case which was cystic partially differentiated nephroblastoma. Staging details were available for three patients. Two were classified as stage 1 and one as stage IV and managed accordingly. Largest series of eight patients of Wilms tumor has been reported from Finland in a cohort of 101 cases of MUL but it lacked details of treatment and outcome.^19^ The treatment details were available for six cases. They were managed mainly with surgery, chemotherapy with/without radiotherapy, and all are in remission. The longest survivor was 15 years at the time of reporting.

**TABLE 1:** Cases of Wilms tumor and Mulibrey Nanism syndrome

Sr. No.	Publication	No. of cases	Age (months)	Sex	Histopathology of WT	Stage	Treatment	Chronology of diagnosis of MUL and WT: Synchronous/MUL before WT/WT before MUL	Other features	Disease status
1	Simila et al.^6^	1	24	F	NA	NA	Chemotherapy (Actinomycin‐D, VCR) + surgery + RT	Synchronous	Nil	Remission
2	Seemanova et al.^15^	1	18	M	NA	NA	Chemotherapy + surgery	WT before MUL	Bone dysplasia/Constrictive pericarditis/Inguinal Hernia/Hypotonia/Ventriculomegaly	Remission
3	Hämäläinen et al.^16^	1	18	F	NA	IV	27 Weeks chemotherapy (Actinomycin‐D, VCR and Doxorubicin) + surgery @ 6 weeks + RT (10 gray)	Synchronous	Bone Dysplasia	Remission
4	Husova et al.^17^	1	18	M	NA	NA	Chemotherapy + surgery	WT before MUL	Constrictive pericarditis, ascites	Remission
5	Seppo Taskinen et al.^18^	1	7	F	Cystic partially differentiated nephroblastoma	I	Chemotherapy (Actinomycin‐D, VCR) + Surgery	WT before MUL	Simple renal cyst in contralateral kidney	Remission
6	Sivunen et at.^19^	8	30 (mean)	NA	NA	NA	NA	MUL before WT	NA	NA
7	Present Study	1	24	M	Triphasic nephroblastoma favorable histology	I	Pre surgery—VCR, Actinomycin‐D and cyclophosphamide (5 weeks) Post‐surgery—VCR and Actinomycin‐D (5 weeks)	Synchronous	ASD, Benign nodules in liver and Simple renal cyst in contralateral kidney	Remission

Abbreviations: ASD, atrial septal defect; F, female; M, male; MUL, Mulibrey Nanism; NA, information not available; RT, radiotherapy; VCR, vincristine; WT, Wilms tumor.

MUL is associated with an increased frequency of benign and malignant tumors and also shows disturbed control of organ development. Karlberg et al. noted a total of 210 tumorous lesions in 66/89 Finnish patients (74%). The benign tumors included cysts, vascular abnormalities (peliosis), adrenal adenoma, parathyroid adenoma, thyroid goiter, pancreatic cystadenoma, renal angiomyolipoma, ovarian fibro thecoma and phaeochromocytoma. Thirteen of the 89 patients (15%) had a malignant tumor, such as WT, renal papillary carcinoma, thyroid papillary and medullar carcinoma, ovarian and endometrial carcinoma, acute lymphoblastic leukemia, Langerhans cell histiocytosis and carcinoid tumor.^20^

WT can be the first presentation, with MUL being diagnosed several years later as seen in three cases found in this review of literature. Children with WT should be assessed for possible syndromic association and be managed accordingly.

Two out of 14 patients initially treated for WT, required pericardiectomy several years later. Our case also has early signs of pericardial thickening and may later require cardiac intervention. Although cardiac affliction is known, arrhythmia as a predominant symptom seen in our patient has not been described previously. Other features seen in patients associated with MUL and WTs included bone dysplasia, constrictive pericarditis requiring pericardiectomy, and massive ascites requiring repeated abdominocentesis.

## 5 DISCUSSION

Our patient presented with WT. In view of child not tolerating chemotherapy well, having abnormal facies, cardiac anomaly, hepatomegaly and abnormal findings in MRI brain; it was suspected that child has some underlying syndrome thus genetic testing was performed. Our patient showed three major signs (small for gestational age lacking catchup growth, craniofacial features, shallow and J‐shaped Sella turcica) and two minor signs (peculiar high‐pitched voice and hepatomegaly) as described by Karlberg et al., depicted in Table 2.^21^ Finland accounts for the maximum number of MUL cases.^22^ MUL can resemble closely to 3M syndrome.^23^

**TABLE 2:** Diagnostic criteria for Mulibrey Nanism and the diagnostic findings in our patient

Signs	Our patient
Major signs	
Growth Failure(A or B or C)	
(A) Small for Gestation Age (SGA) lacking catch up growth	√
(B) Height in Children 2.5 below population mean for age	
(C) Height in adult 3.0 SD below population mean	
Characteristic Radiological Findings (A or B)	
(A) Slender long bones with thick cortex and narrow medullary channel	
(B) J‐shaped Sella turcica	√
Characteristics craniofacial features Scaphocephaly, triangular face, high and broad forehead, low nasal bridge and telecanthus	√
Characteristic ocular findings	
Yellowish dots in Retinal mid peripheral region	
Mulibrey Nanism in Sibling	
Minor signs	
Peculiar High pitched Voice	√
Hepatomegaly	√
Cutaneous Naevi Flammei	
Fibrous Dysplasia of Long Bone	

*Note*: For the diagnosis, three major signs and one minor sign or two major signs with three minor signs are required.

MUL is associated with an increased frequency of benign and malignant tumors and also shows disturbed control of organ development. The association of TRIM37 mutations with increased risk of cancer indicates a pro‐survival process in TRIM37‐deficient tumor cells. Wang et al. reported interaction of lysosomal protein mTOR with TRIM37, leading to transcriptional activation of genes involved in lysosome biogenesis and macro autophagy/autophagy. Autophagy could be a way for the cell to survive the loss of TRIM37.^24^ In several non‐MUL cancers, TRIM37 has been found to be overexpressed. TRIM37 plays a critical role in cell proliferation and, therefore its overexpression has been detected in a variety of human tumors.^25^ The decrease in TRIM37 results in loss of ubiquitinated H2A and therefore a decrease in tumor growth, while its overexpression induces tumorigenesis.^26^Although WT has been previously reported in MUL patients, cases of familial WT associated with TRIM37 outside of MUL have not been documented.

The missense variation in exon 2 of the TRIM37 gene variant *c*.*80T>A* noted in our patient was novel and has not been reported previously.^25^ This mutation was also found in the father and hence possibly can be considered to confer pathological attributes. The other mutation found in the patient in exon 15 of the TRIM37 gene variant *c*.*1336C>T* is a reported pathogenic variant and is probably de novo acquired in our patient.

The coexisting liver lesion which was initially considered as hepatic metastasis and was later diagnosed as benign liver hamartomas, emphasizes the need to look out for associated benign tumors. These patients are highly prone to tumorigenesis and careful diagnosis can help in assigning the correct stage and formulating relevant treatment protocol.

Heart is the most frequently affected organ in MUL with constrictive pericarditis, myocardial hypertrophy and variable myocardial fibrosis constituting the main components of Mulibrey heart disease. Mariata et al. reported 49 adults with MUL heart disease, noted these patients to be highly susceptible to develop congestive cardiac failure and need for pericardiectomy in one third of the patients.^27^ ASD similar to our case have been reported in MUL.^28^, ^29^ Our patient had two episodes of AF and an episode of sudden cardiac arrest which has not been reported previously in children with MUL. Structural heart defect, particularly ASD especially ostium primum is known to be associated with arrhythmias, with increasing frequency with age.^30^ Cases of resistant to treat arrhythmia have been managed successfully with ASD closure in adults, but reports in children are limited. Our patient responded to anti‐arrhythmic drugs. The temporal proximity of AF and COVID‐19 infection might raise questions if the AF was due to COVID‐19. However, the first episode of atrial flutter happened about 1 week before the patient was tested positive for COVID‐19 as depicted in Figure 1. Atrial flutter is not a known side‐effect of the chemotherapy that the patient had received. Most likely underlying cardiac defect contributed to the episodes of atrial flutter.

WT although rare in children with MUL, is treatable. Our case highlights the need to watch out for cardiac arrhythmia in these patients. Although a lot could not be gathered form review of literature regarding treatment protocol, but it appears that these children tolerate standard treatment protocol well and can achieve remission.

## CONFLICT OF INTEREST

All authors have nothing to disclose.

## AUTHOR CONTRIBUTIONS

All authors had full access to the data in the study and take responsibility for the integrity of the data and the accuracy of the data analysis. *Conceptualization*, K.U., D.T., M.S.S., A.Y., K.B., J.S., N.R., S.P.Y.; *Methodology*, K.U., D.T., D.G., M.S.S., A.Y., R.K., V.R., K.B., S.N., J.S., N.R., S.P.Y.; *Investigation*, K.U., D.T., M.S.S., R.K., V.R., M.S.D., K.B., S.N., J.S., N.R., S.P.Y.; *Formal Analysis*, K.U., M.S.S., A.Y., R.K., V.R., K.B., S.N., J.S., N.R., S.P.Y.; *Resources*, K.U., D.T., D.G., M.S.S., A.Y., V.R., M.S.D., S.N., J.S., N.R., S.P.Y.; *Writing—Original Draft*, K.U., D.G., J.S., N.R., S.P.Y.; *Writing—Review & Editing*, D.T., M.S.S., R.K., K.B., N.R., S.P.Y.; *Visualization*, K.U., D.T., M.S.S., A.Y., R.K., V.R., M.S.D., K.B., S.N., J.S., N.R., S.P.Y.; *Supervision*, R.K., V.R., K.B., N.R., S.P.Y.; *Funding Acquisition*, D.T., A.Y., R.K., K.B., S.N., J.S., N.R., S.P.Y.; *Data Curation*, K.U., D.T., D.G., M.S.S., A.Y., R.K., V.R., M.S.D., K.B., S.N., J.S., N.R., S.P.Y.; *Project Administration*, K.U., D.T., K.B., N.R., S.P.Y.; *Software*, K.U., D.T., D.G., M.S.S., A.Y., V.R., K.B., S.N., J.S., N.R., S.P.Y.; *Validation*, K.U., D.T., A.Y., R.K., V.R., K.B., S.N., J.S., N.R., S.P.Y.

## ETHICAL STATEMENT

Institutional approval N/A. Informed consent of parent obtained.

## Data Availability

Data sharing is not applicable to this article as no new data were created or analyzed in this study.
